# Additive effects of simulated microgravity and ionizing radiation in cell death, induction of ROS and expression of RAC2 in human bronchial epithelial cells

**DOI:** 10.1038/s41526-020-00123-7

**Published:** 2020-11-05

**Authors:** Shaobo Tan, Weiwei Pei, Hao Huang, Guangming Zhou, Wentao Hu

**Affiliations:** State Key Laboratory of Radiation Medicine and Protection, School of Radiation Medicine and Protection, Collaborative Innovation Center of Radiological Medicine of Jiangsu Higher Education Institutions, Soochow University, Suzhou, 215123 China

**Keywords:** Biophysics, Risk factors

## Abstract

Radiation and microgravity are undoubtedly two major factors in space environment that pose a health threat to astronauts. However, the mechanistic study of their interactive biological effects is lacking. In this study, human lung bronchial epithelial Beas-2B cells were used to study the regulation of radiobiological effects by simulated microgravity (using a three-dimensional clinostat). It was found that simulated microgravity together with radiation induced drop of survival fraction, proliferation inhibition, apoptosis, and DNA double-strand break formation of Beas-2B cells additively. They also additively induced Ras-related C3 botulinum toxin substrate 2 (RAC2) upregulation, leading to increased NADPH oxidase activity and increased intracellular reactive oxygen species (ROS) yield. The findings indicated that simulated microgravity and ionizing radiation presented an additive effect on cell death of human bronchial epithelial cells, which was mediated by RAC2 to some extent. The study provides a new perspective for the better understanding of the compound biological effects of the space environmental factors.

## Introduction

With the development of aerospace technology, astronauts spend more time in the space environment and the threats to the life and health of astronauts from space radiation become more serious^1,2^. However, the space environment is a multi-factor compounding field and the biological effects of space radiations are affected by a variety of space environmental factors, such as microgravity, weak magnetic fields, circadian rhythm changes, etc., which makes it difficult to demonstrate the biological effects of space radiations and increases the uncertainty of space radiation risk assessment^3–6^.

As an inevitable space environmental factor, the effect of microgravity on the biological effects of radiation has received widespread attention^7^. Girardi et al.^8^ studied the expression profiles of both miRNAs and mRNAs in human peripheral blood lymphocytes irradiated with γ-rays under simulated microgravity (SMG) conditions, and found that microgravity affects the radiation-induced DNA damage response pathway through bioinformatics analysis. Pani et al.^1^ found that radiation and SMG can synergistically reduce neural network integrity and nerve cell survival. Dang et al.^9^ found that SMG aggravates human B-lymphocyte apoptosis induced by heavy ion radiation. However, research on the regulation of SMG on radiobiological effects is still very limited. The underlying mechanism by which microgravity regulates radiobiological effects remains unclear.

Previous studies based on epidemiology from both astronauts and atomic bomb survivor data have determined radiation-induced lung cancer as the greatest carcinogenic risk for solid tumors^10,11^. Besides, our previous study showed that Ras-related C3 botulinum toxin substrate 2 (RAC2) plays an important role in cellular radiobiological effects^12^. In this study, we investigated the effects of both SMG and ionizing radiation (IR) on the survival, proliferation, apoptosis, and DNA double-strand breaks damage of human bronchial epithelial Beas-2B cells, and revealed the combined biological effects of SMG and radiation are related to RAC2 expression, NADPH oxidase activity, and reactive oxygen species (ROS) yield.

## Results

### Both SMG and X-ray radiation induced cell survival inhibition

**Fig. 1:**
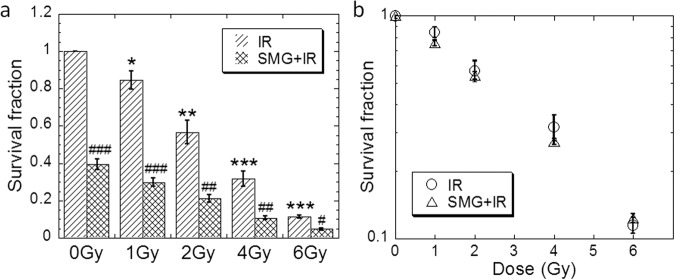
Both simulated microgravity and X-ray irradiation induced cell survival inhibition. Beas-2B cells were treated by simulated microgravity for 48 h first and then exposed to X-ray irradiation at indicated doses. **a** Cell survival fraction was determined by colony formation assay. **b** Cell survival curves were drawn for cells subjected to irradiation and/or simulated microgravity. The experiments were done in triplicate and error bars indicate SEM. IR, irradiation group; SMG, simulated microgravity group; SMG + IR, simulated microgravity combined with irradiation group. For the compounding treatment, cells were treated by simulated microgravity for 48 h first and then exposed to X-ray irradiation. Cells were cultured under 1 g during and after exposure (the same below). *Compared with control group. #Compared with IR group, */^#^*p* < 0.05, **/##*p* < 0.01, ***/###*p* < 0.001.

First, we investigated the effect of the SMG and X-ray irradiation on cell survival of human lung epithelial Beas-2B cells. As shown in Fig. 1, we found that SMG significantly reduced the cell survival level even in the unirradiated group (*p* < 0.001) (Fig. 1a). As for cells irradiated with the indicated doses of X-rays, IR inhibited the cell survival significantly at all the gradient doses from 1 Gy through 6 Gy (*p* < 0.05 at all dose points) (Fig. 1a), whereas the SMG and X-ray irradiation (SMG + IR) induced cell survival inhibition additively at all doses we tested as indicated by the similar survival curves (Fig. 1b).

### Both SMG and radiation induced cell proliferation inhibition

**Fig. 2:**
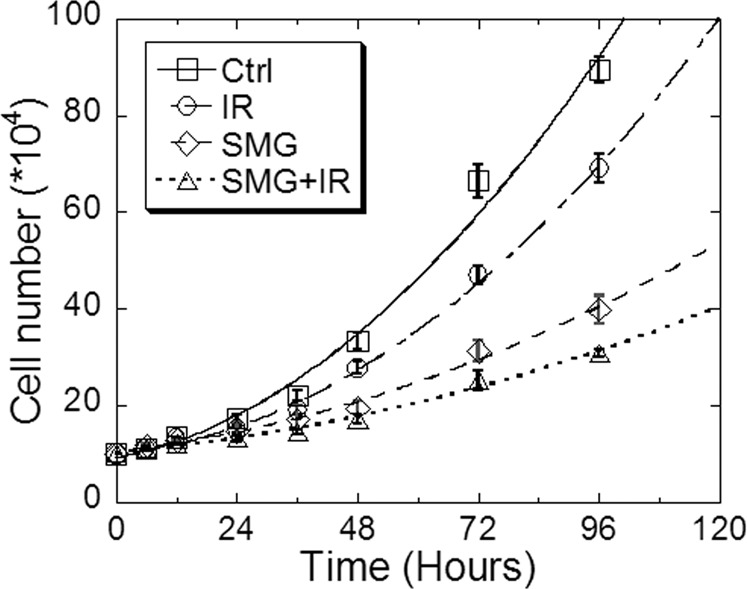
Both simulated microgravity and radiation induced cell proliferation inhibition. Beas-2B cells were treated by simulated microgravity for 48 h first and then exposed to 2 Gy X-ray irradiation. Cell proliferation curves were drawn from 0 to 96 h post irradiation. The experiments were done in triplicate and error bars indicate SEM. Ctrl, control group; IR, irradiation group; SMG, simulated microgravity group; SMG + IR, simulated microgravity combined with irradiation group.

Then the cell proliferation curves were drawn for Beas-2B cells subjected to different treatments: control group (Ctrl), irradiation group (IR), SMG group, and compounding treatment group (SMG + IR) from 0 to 96 h post irradiation (Fig. 2). We found that SMG treatment alone can induce the inhibition of cell proliferation. From 48 h, the SMG inhibited cell proliferation even more strongly than 2 Gy X-ray radiation, whereas both factors showed an additive effect on the inhibition of cell proliferation.

### Effects of SMG and radiation on DNA double-strand break formation

**Fig. 3:**
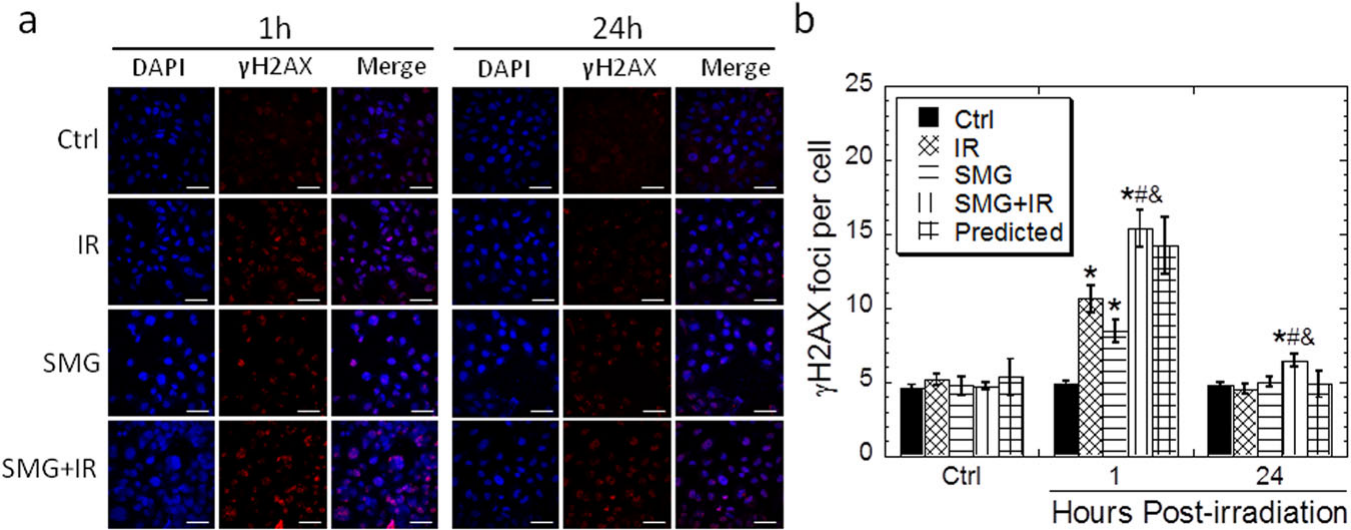
The γH2AX foci levels in the Beas-2B cells subjected to different treatments. Beas-2B cells were treated by simulated microgravity for 48 h first and then exposed to 0.5 Gy X-ray irradiation. Irradiated cells were fixed at 1 h or 24 h post irradiation for γH2AX foci assay. **a** Immunofluorescent staining of the γH2AX in the cells exposed to simulated microgravity and/or 0.5 Gy X-rays. **b** The γH2AX foci yields in the cells exposed to simulated microgravity and/or 0.5 Gy X-rays. The experiments were done in triplicate and error bars indicate SEM. Ctrl, control group; IR, irradiation group; SMG, simulated microgravity group; SMG + IR, simulated microgravity combined with irradiation group. Scale bar represents 50 μm. *Compared with control group. #Compared with IR group. &Compared with SMG group. */#/&: *p* < 0.05.

Radiation-induced DNA double-strand breaks are an important cause of cell death subjected to radiation. Thus, γH2AX foci assay was employed to evaluate the effect of SMG on DNA double-strand breaks in Beas-2B cells irradiated by 0.5 Gy X-rays. As shown in Fig. 3, SMG treatment alone induced the formation of γH2AX foci significantly (*p* < 0.05). SMG combined with X-ray radiation (SMG + IR) significantly promoted the formation of γH2AX foci when compared to microgravity/radiation alone (SMG/IR) (*p* < 0.05). Twenty-four hours after irradiation, γH2AX foci in the cells of the SMG/irradiation (SMG/IR) returned to the control level (*p* > 0.05); however, it did not recover in the compounding treatment group (SMG + IR) and was significantly higher than control level (Ctrl) (*p* < 0.05). To determine whether there exists a synergistic effect between SMG and irradiation for γH2AX foci induction, we presented the predicted γH2AX foci yield based on the additive effects^13^. SMG alone induced about 3.59 foci per cell compared to the control. The predicted number based on additive effects was the sum of 3.59 and the number of foci per cell for irradiated cells. As shown in Fig. 3, the γH2AX foci yield in cells after combined treatment agreed well with the prediction, suggesting it is an additive effect rather than a synergistic effect that exists between SMG and irradiation for γH2AX foci induction.

### SMG and radiation induced apoptosis additively

**Fig. 4:**
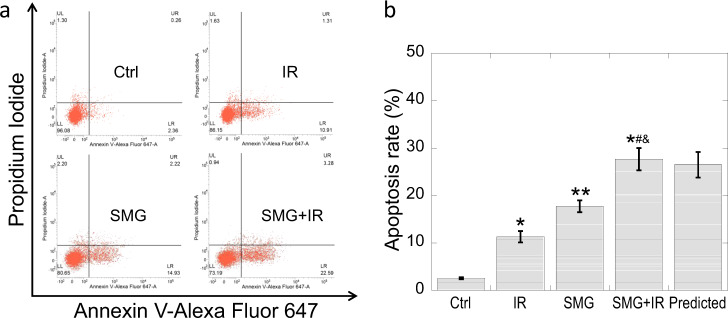
Simulated microgravity and radiation induced apoptosis additively. Beas-2B cells were treated by simulated microgravity for 48 h first and then exposed to 2 Gy X-ray irradiation. Samples were collected at 48 h post irradiation. **a** Representative images of the cytometric analysis of the apoptosis. **b** The apoptosis rates of the cells exposed to simulated microgravity and/or 2 Gy X-rays. The experiments were done in triplicate and error bars indicate SEM. Ctrl, control group; IR, irradiation group; SMG, simulated microgravity group; SMG + IR, simulated microgravity combined with irradiation group. *Compared with control group. #Compared with IR group. &Compared with SMG group. */#/&: *p* < 0.05, **: *p* < 0.01.

We used Annexin V-Alexa Fluor 647/PI dual-staining apoptosis detection kit to detect the apoptosis levels in Beas-2B cells with different treatments and found that SMG induced significant apoptosis (*p* < 0.05). The apoptosis level in SMG group was even higher than that in 2 Gy X-ray-irradiated group (IR) (*p* < 0.05) and the combination of SMG and 2 Gy X-ray irradiation (SMG + IR) induced more apoptosis when compared to SMG/radiation alone (SMG/IR) (*p* < 0.05) (Fig. 4). Based on the interactive effect analysis mentioned above, an additive effect was also found between SMG and irradiation for apoptosis induction.

### SMG and radiation increased RAC2 expression, NADPH oxidase activity, and ROS yield additively

**Fig. 5:**
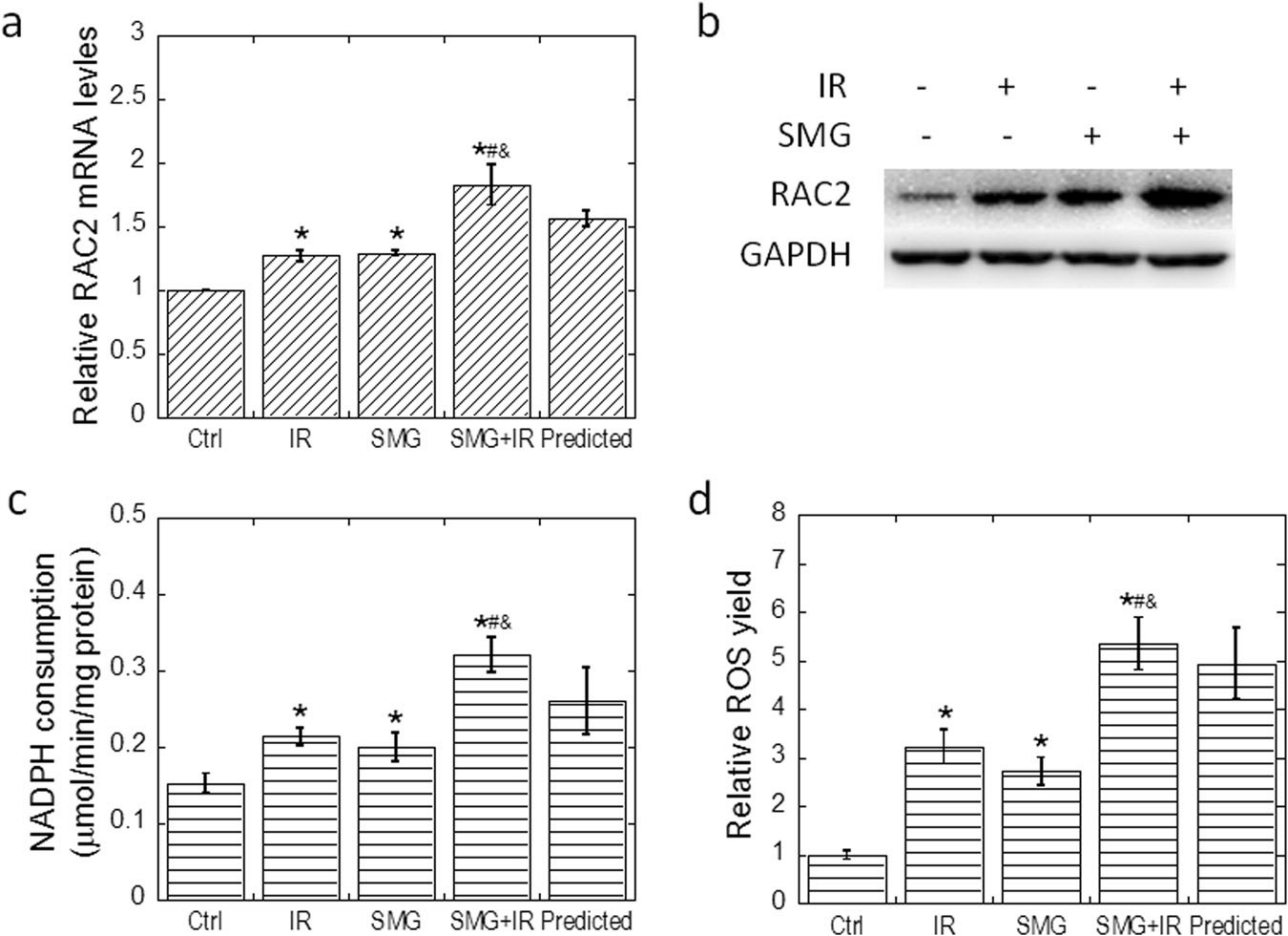
Simulated microgravity and radiation increased RAC2 expression, NADPH oxidase activity, and ROS yield additively. Beas-2B cells were treated by simulated microgravity for 48 h first and then exposed to 2 Gy X-ray irradiation. Samples for qRT-PCR, western blotting, NADPH consumption assay, as well as ROS production assay were all collected at 2 h post irradiation. **a** Transcriptional level of RAC2 was determined in Beas-2B cells exposed to simulated microgravity and/or 2 Gy X-rays. **b** Protein level of RAC2 as determined by western blotting. **c** NADPH consumption in Beas-2B cells exposed to simulated microgravity and/or 2 Gy X-rays. **d** ROS yields in the treated Beas-2B cells. All experiments were done in triplicate and error bars indicate SEM. Ctrl, control group; IR, irradiation group; SMG, simulated microgravity group; SMG + IR, simulated microgravity combined with irradiation group. *Compared with control group. #Compared with IR group. &Compared with SMG group. */#/&: *p* < 0.05.

In our previous research, we found that RAC2 is involved in modulating the radiosensitivity of cells, which is the main intracellular subunit that activates NADPH oxidase and promotes the production of ROS. ROS is a decisive molecule for radiation-induced cell death and plays a key role in signal transduction. Therefore, we tested the effect of SMG and/or irradiation on RAC2 expression in Beas-2B cells. Quantitative real-time PCR data showed that either 2 Gy X-ray irradiation (IR) or SMG induced RAC2 expression significantly, whereas the induction effect of compounding treatment (SMG + IR) was more pronounced than that of single factor treatment (SMG/IR) (*p* < 0.05, Fig. 5a). Western blotting experiments also confirmed the finding of the quantitative PCR (Fig. 5b). NADPH oxidase activity and ROS yield measurements indicated that the increased RAC2 expression promoted the increase of consequent NADPH oxidase activation and ROS production (Fig. 5c, d). The interactive effects between SMG and irradiation for induction of RAC2 expression, NADPH oxidase activity, as well as ROS production were also analyzed by the method mentioned above. As shown in Fig. 5a, c, d, additive effects of SMG and irradiation for the induction of RAC2 expression, NADPH oxidase activity, as well as ROS production were observed.

## Discussion

Microgravity and space radiation have been proved to have a variety of detrimental effects on human health^7,14^. However, the biological effects of only one of them are studied in most research and there is still a lack of sufficient understanding for the compound biological effect of the two major space environmental factors. Besides, most studies of the compound biological effect are limited to phenomenon description^8,9,15^. The underlying mechanisms of the compounding effects of the two detrimental factors remain elusive. Lung cancer is the biggest health threat for both male and female astronauts in deep space exploration according to the NASA’s study^10^. However, the compound biological effects of SMG and IR on the lung cells have not been studied yet. Our study found that the SMG and IR presented additive effects on the induction of cell death, as well as NADPH oxidase activity and the consequent ROS production in human bronchial epithelial cells.

According to our previous research, RAC2 is an important cytosolic subunit of the NADPH oxidase and responsible for its activation and production of ROS^12^. ROS, as an important mediator of radiobiological effects, functions in the process of DNA damage caused by IR and is the decisive molecule of radiation-induced cell death^16,17^. Referring to the results of this study, RAC2 was induced upon either SMG or radiation exposure treatment, and the both showed an additive effect. RAC2 expression level was found to be positively correlated with ROS production, NADPH oxidase activity, and DNA double-strand breaks formation in Beas-2B cells treated with SMG and/or radiation, indicating that RAC2 played an important role in the compound biological effects of microgravity and radiation. Besides, it was reported that treatment with SMG alone could increase ROS production and cause DNA damage^7,18,19^, which is in accordance with our results. However, our research first connects this with the increased RAC2 expression and NADPH oxidase activity. Further, RAC2 is also shown to be implicated in the polarization of epithelial cells, which is a process closely related to the changes of intercellular junctions, cytoskeleton distribution, organelle repositioning, etc.^20,21^. The shape and structure changes of cells might also enhance the effects caused by SMG. The underlying mechanisms of RAC2-related polarization in epithelial cells subjected to SMG deserve further research.

As the major space environmental factors, both microgravity and space radiation have been reported to cause a variety of biological effects, such as micronuclei formation, gene expression, DNA damage response, apoptosis, chromosome aberration, etc.^7,14,22^. However, most of these studies focused on whether the radiobiological effects could be affected by microgravity due to the paradigm that space radiation-induced DNA damage is considered as a decisive factor for the fate of the cell. In our study, RAC2 was found to be induced by SMG independently and the resulting increased ROS production resulted in both DNA damage and cell death, implying that both microgravity and radiation interact at the initial step of signal transduction pathways mediated by ROS.

The results of this study provide a new perspective for the study of the mechanism of multi-factor biological effects in space environment and are of great significance for the study of the mechanisms underlying the combined biological effects of microgravity and radiation. It should be noted that, the gravity of objects is difficult to be offset apart from free fall and earth orbit motion; thus, it is difficult to simulate microgravity environment in general laboratory. In light of this, some kinds of equipment, such as clinostat, were used to simulate the effect of microgravity to some extent. The clinostat is a device for rotating the biological samples around one or several axes. The magnitude of the gravity vector changes continuously to the relative direction of the sample and the vector sum turns out to be zero for each rotation cycle, which is equivalent to the microgravity state. For the studies carried out with clinostats, the term “simulated microgravity” is used to describe the resulting state of clinorotation. Besides, as the radiation used in our study is X-rays of low LET (linear energy transfer), further studies employing high atomic number and high energy radiations are needed to demonstrate the effects of the SMG on the biological effects of the space IR.

## Methods

### Cell culture

Human bronchial epithelial cell line Beas-2B was purchased from American Type Culture Collection (CRL-9609) and maintained in Dulbecco’s modified Eagle’s medium (Gibco, Grand Island, NY, USA) containing 10% fetal bovine serum (Gibco, Grand Island, NY, USA), penicillin (100 U/mL), and streptomycin (100 µg/mL) in a fully humidified incubator with 5% CO_2_ at 37 °C (Thermo Fisher Scientific, NC, USA).

### Irradiation and SMG

A three-dimensional (3D) clinostat (SM-31, Center for Space Science and Applied Research, Chinese Academy of Sciences) was used to simulate microgravity at 37 °C for 48 h at a random rotating speed of 0~10 r.p.m.^23^. For the immunofluorescence assay, the cells were seeded in the chamber slides (Thermo Scientific, USA) filled up with the medium. The chamber slide was sealed with parafilm before loading on the 3D clinostat. For other experiments, the cells were seeded in T12.5 culture flasks (Corning, USA) filled up with medium and sealed with parafilm before SMG treatment. The clinostat with cell culture vessels was placed in a humidified incubator (5% CO_2_, 37 °C) and was connected to the control console outside of the incubator. X-ray irradiation was performed at room temperature in a RS 2000 X-ray Biological Irradiator (Rad Source Technologies, Suwanee, GA, USA) at a dose rate of 1.2 Gy/min. The energy of X-rays was 100 kVp. For the compounding treatment with both SMG and X-ray irradiation, cells were treated by SMG for 48 h first and then exposed to X-ray irradiation. Cells were cultured under 1 g during and after radiation. The astronauts will receive a total estimated dose equivalent of 331 ± 54 mSv from galactic cosmic radiation (GCR) during a one-way trip to Mars^24^. Thus, in our study, 2 Gy X-rays, which is equivalent to about 0.4 Gy Fe ions in cell killing of human lung epithelial cells (the range of RBEs for ^56^Fe is from 3.91 to 5.92 as reported by Ding et al.^25^), was used for the most endpoints detected in our study.

### Colony formation assay

Beas-2B cells were harvested immediately after irradiation by trypsinization, then counted, and plated into Φ60 mm dishes with 5 mL medium (preheated to 37 °C) and returned to the incubator to produce 20–100 colonies. After growing for 14 days, cells were fixed with 70% ethanol for 5 min and stained with 0.5% crystal violet. Colonies containing more than 50 cells were counted as survivors. At least three parallel dishes were scored for each treatment.

### Cell proliferation

The treated cells were collected and counted, and the cells were seeded in a 12-well plate at 100,000 cells/well, and cultured in a 37 °C, 5% CO_2_ saturated humidity incubator. Cells in the wells were counted after 0, 6, 12, 24, 36, 48, 72, and 96 h, respectively. Three parallel wells were set in each group.

### γH2AX foci analysis

Samples were collected at 1 and 24 h after irradiation. After removing the culture medium, the cells were washed with phosphate-buffered saline (PBS), fixed with 4% paraformaldehyde for 10 min, and fixed with iced methanol for another 10 min, permeabilized with 0.1% Triton X-100 for 5 min, blocked with 5% skim milk for 1 h, and then incubated with mouse anti-γH2AX antibody (Cell Signaling Technology, Beverly, MA, USA) for 4 h at room temperature. After washed by PBST (PBS with 0.1% Tween-20) for three times, the cells were hybridized for 1 h with secondary antibody labeled with Alexa Fluor 555. After washing with PBST for five times and 4′,6-diamidino-2-phenylindole staining, foci were counted under a laser scanning confocal microscope (Olympus FV1200, Tokyo, Japan). At least 100 cells were counted for each sample.

### Cell apoptosis assay

The treated Beas-2B cells were collected, washed twice with pre-cooling PBS at 4 °C, and resuspended with 250 μL binding buffer to adjust the concentration to 1 × 10^6^ cells/mL. Then cells were stained with Annexin V-Alexa Fluor 647/PI Apoptosis Detection Kit (Fcmacs, Nanjing, China). Briefly, 5 μL Annexin V/Alexa Fluor 647 and 10 μL propidium iodide solution (20 μg/mL) were added into 100 μL of cell suspension in a 5 mL flow tube, mixed, and incubated at room temperature for 15 min in the dark, and 400 μL PBS was then added into the tube. Cell apoptosis was analyzed with a FACSVerse flow cytometer (BD Biosciences, Franklin Lakes, NJ, USA).

### Quantitative reverse transcriptase PCR

Cells were collected in TRIzol reagent (Invitrogen, CA, USA). Total RNAs were reverse-transcribed using PrimeScript RT Reagent Kit (Takara, Kusatsu, Shiga, Japan). A PCR analysis was performed using PowerUp™ SYBR^®^ Green Master Mix (Life Technologies, Grand Island, NY, USA) and amplified PCR products were quantified and normalized with glyceraldehyde 3-phosphate dehydrogenase (GAPDH). The PCR amplification was carried out using a Life Technologies system (Vii7A, NY, USA) and initiated by 2 min at 95 °C before 40 thermal cycles, 30 s each at 95 °C, and 40 s at 62 °C with a final extension of 10 min at 72 °C. Data were analyzed by C(t) value comparison method and normalized by control expression in each sample. The primer sequences for RAC2 amplification were as follows: forward: 5′-CGCCAAGTGGTTCCCAGAAG-3′, reverse: 5′-AGCTGAGCACTCCAGGTATTT-3′. The GAPDH primer sequences were as follows: forward: 5′-GCACCGTCAAGGCTGAGAAC-3′, reverse: 5′-TGGTGAAGACGCCAGTGGA-3′.

### Immunoblotting

Cells were lysed using RIPA buffer (Beyotime, Nantong, China). Samples were sonicated and centrifuged at 13,523 × *g* for 15 min at 4 °C. The total protein concentration was determined by using a DC Protein Assay Kit (Bio-Rad, Richmond, CA, USA). Samples were denatured at 100 °C for 5 min, separated using SDS-polyacrylmide gel electrophoresis, and transferred to polyvinylidene difluoride membranes (GE Healthcare, Piscataway, NJ, USA). After blockage with PBST containing 5% skimmed milk for 1 h, the membrane was incubated with primary antibodies for 2 h at room temperature, washed three times with PBST for 5 min each, and then incubated with horseradish peroxidase-conjugated secondary antibody for 1 h and washed three times with PBST. Protein bands were visualized using an ECL kit (Millipore, Billerica, MA, USA). Anti-RAC2 was purchased from Abcam (Cambridge, MA, USA) and anti-GAPDH was from Cell Signaling Technology (Beverly, MA, USA). All blottings derived from the same experiment and were processed in parallel. The uncropped images of all blots are shown in Supplementary Fig. 1.

### Measurement of ROS yield and NADPH oxidase activity

ROS yield and NADPH oxidase activity of the treated cells were measured using the ROS Detection Kit (Beyotime, Shanghai, China) and the NADPH Oxidase Assay Kit (Jiancheng, Nanjing, China) in strict accordance with the manufacturers’ manuals. The corresponding absorbance was detected on a Synergy 2 microplate reader (BioTek Instruments, VT, USA).

### Statistical analysis

Statistical analysis was performed on the means of the data obtained from at least three independent experiments. Data are presented as the means ± SE. All statistical analyses were executed by two-tailed Student’s *t*-test using Excel (Microsoft). *p* < 0.05 was considered as statistically significant. The corresponding data points (as dot plots) were overlaid in the bar charts as shown in Supplementary Fig. 2.

### Reporting summary

Further information on experimental design is available in the Nature Research Reporting Summary linked to this paper.

## Supplementary information

Supplementary information

Reporting Summary Checklist FLAT

## Data Availability

All data generated or analyzed during this study are included in this published article and its Supplementary Information files.
