# Supplementary material for: Additive effects of simulated microgravity and ionizing radiation in cell death, induction of ROS and expression of RAC2 in human bronchial epithelial cells
Source: NPJ Microgravity. 2020 Nov 5;6:34. doi: 10.1038/s41526-020-00123-7 (PMC7645497; doi:10.1038/s41526-020-00123-7)
Supplement: Supplementary file 1 — Supplementary information [file 41526_2020_123_MOESM1_ESM.pdf]

Supplementary Methods

Simulated microgravity. As shown in Supplementary Methods figure 1, the 3D clinostat (SM-31, Center for Space Science and Applied Research, Chinese Academy of Sciences) consists of two rotating frames, outer rotating frame and inner rotating frame driven by independent motors. The inner frame is fixed to the outer frame rotating around the horizontal axis. The two rotating shafts are perpendicular to each other. The speed, direction and duration time are controlled by the controller with single chip microcomputer. There are three kinds of rotation mode: (a) The two frames rotate at the same constant speed (±0~30 rpm) in same/reverse directions; (b) The two frames rotate at different constant speed (±0~30 rpm) in same/reverse directions; (c) The two frames rotate at random speed (±0~30 rpm) in same/reverse directions. From the point of view of the sample, the gravity vector's trajectory averaged over time shall converge toward zero. Consequently, the biological samples will experience a state similar to microgravity. In this study, the random rotation mode with a speed ranging from 0 to 10 rpm was set for both frames. For the immunofluorescence assay, the cells were seeded in the chamber slides (Thermo Scientific, USA) filled up with the medium. The chamber slide was sealed with parafilm before loading on the 3-D clinostat. For other experiments, the cells were seeded in T12.5 culture flasks (Corning, USA) filled up with medium and sealed with parafilm before SMG treatment. 48h of simulated microgravity was carried out only prior to the X-ray irradiation. Cells were under 1 g during and after radiation.

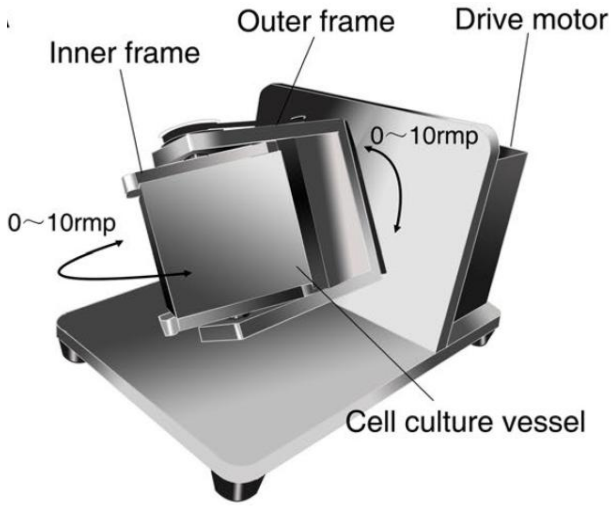

Supplementary Methods figure 1. The schematic map of the 3D clinostat we used.

Set of experimental time points:

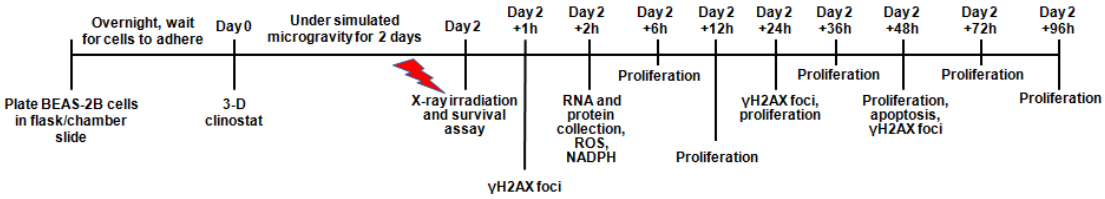

Supplementary Methods figure 2. The sketch map for experimental time point setting in this study.

Supplementary figure 1

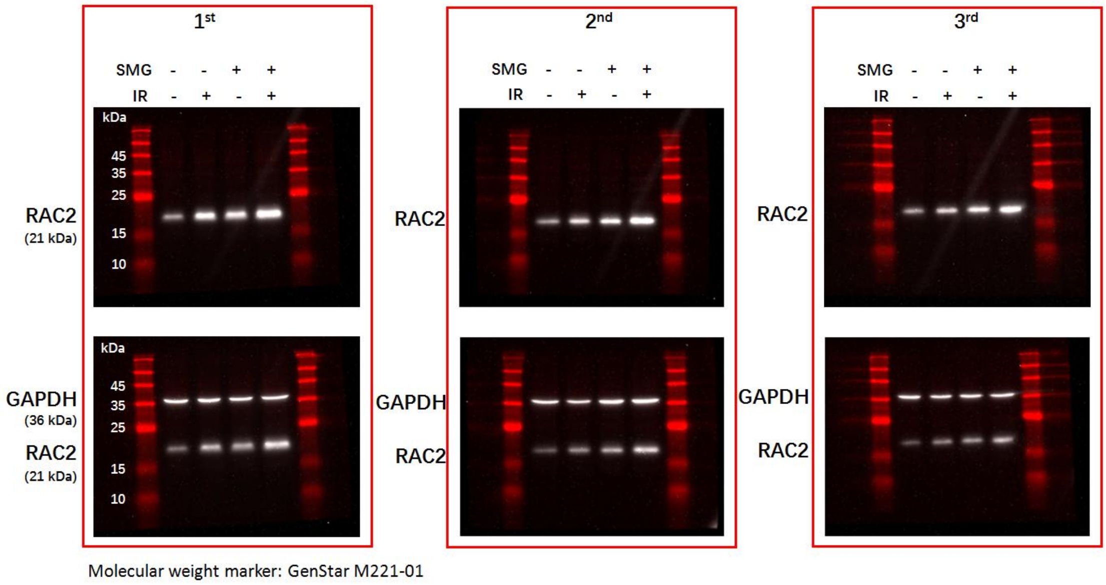

Supplementary figure 1. The uncropped images of all blots. All samples were separated using 15% SDS-PAGE. The wet transfer was run at 150 mA for 1.5 hrs.

Supplementary figure 2

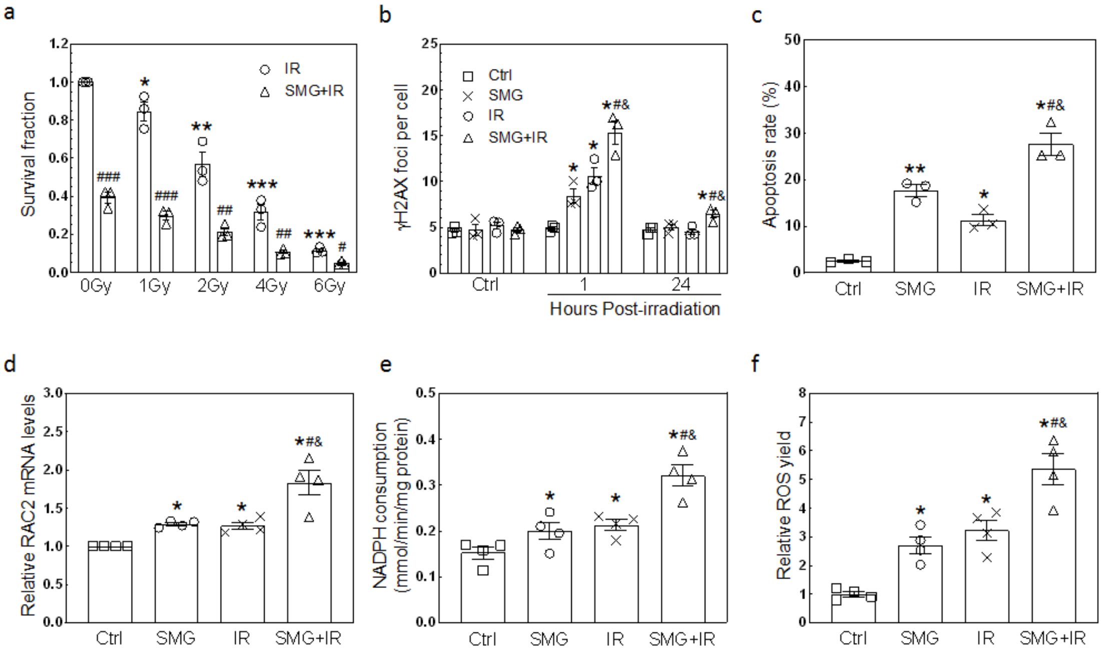

Supplementary figure 2. The corresponding data points (as dot plots) were overlaid in the bar charts including Figure 1a, 3b, 4b, 5a, 5c, 5d. (a) Cell survival fraction was determined by colony formation assay. (b) The γH2AX foci yields in the cells exposed to simulated microgravity and/or 0.5 Gy X-rays. (c) The apoptosis rates of the cells exposed to simulated microgravity and/or 2 Gy X-rays. (d) Transcriptional level of RAC2 was determined in Beas-2B cells exposed to simulated microgravity and/or 2 Gy X-rays. (e) NADPH consumption in Beas-2B cells exposed to simulated microgravity and/or 2 Gy X-rays. (f) ROS yields in the treated Beas-2B cells.
