# Supplementary material for: Additive effects of simulated microgravity and ionizing radiation in cell death, induction of ROS and expression of RAC2 in human bronchial epithelial cells
Source: NPJ Microgravity. 2020 Nov 5;6:34. doi: 10.1038/s41526-020-00123-7 (PMC7645497; doi:10.1038/s41526-020-00123-7)
Supplement: Supplementary file 2 — Reporting Summary Checklist FLAT [file 41526_2020_123_MOESM2_ESM.pdf]

nature research

Corresponding author(s): Wentao Hu

Last updated by author(s): Sep 4, 2020

Reporting Summary

Nature Research wishes to improve the reproducibility of the work that we publish. This form provides structure for consistency and transparency in reporting. For further information on Nature Research policies, see Authors & Referees and the Editorial Policy Checklist.

Statistics

For all statistical analyses, confirm that the following items are present in the figure legend, table legend, main text, or Methods section.

n/a	Confirmed	
☐	☒	The exact sample size (*n*) for each experimental group/condition, given as a discrete number and unit of measurement
☐	☒	A statement on whether measurements were taken from distinct samples or whether the same sample was measured repeatedly
☐	☒	The statistical test(s) used AND whether they are one- or two-sided<br>*Only common tests should be described solely by name; describe more complex techniques in the Methods section.*
☒	☐	A description of all covariates tested
☒	☐	A description of any assumptions or corrections, such as tests of normality and adjustment for multiple comparisons
☐	☒	A full description of the statistical parameters including central tendency (e.g. means) or other basic estimates (e.g. regression coefficient) AND variation (e.g. standard deviation) or associated estimates of uncertainty (e.g. confidence intervals)
☐	☒	For null hypothesis testing, the test statistic (e.g. *F*, *t*, *r*) with confidence intervals, effect sizes, degrees of freedom and *P* value noted<br>*Give P values as exact values whenever suitable.*
☒	☐	For Bayesian analysis, information on the choice of priors and Markov chain Monte Carlo settings
☒	☐	For hierarchical and complex designs, identification of the appropriate level for tests and full reporting of outcomes
☒	☐	Estimates of effect sizes (e.g. Cohen's *d*, Pearson's *r*), indicating how they were calculated

Our web collection on statistics for biologists contains articles on many of the points above.

Software and code

Policy information about availability of computer code

Data collection	*Provide a description of all commercial, open source and custom code used to collect the data in this study, specifying the version used OR state that no software was used.*
Data analysis	*Provide a description of all commercial, open source and custom code used to analyse the data in this study, specifying the version used OR state that no software was used.*

For manuscripts utilizing custom algorithms or software that are central to the research but not yet described in published literature, software must be made available to editors/reviewers. We strongly encourage code deposition in a community repository (e.g. GitHub). See the Nature Research guidelines for submitting code & software for further information.

Data

Policy information about availability of data

All manuscripts must include a data availability statement. This statement should provide the following information, where applicable:

- Accession codes, unique identifiers, or web links for publicly available datasets
- A list of figures that have associated raw data
- A description of any restrictions on data availability

All relevant data are available from the corresponding author.

Field-specific reporting

Please select the one below that is the best fit for your research. If you are not sure, read the appropriate sections before making your selection.

☒ Life sciences ☐ Behavioural & social sciences ☐ Ecological, evolutionary & environmental sciences

Life sciences study design

All studies must disclose on these points even when the disclosure is negative.

Sample size	More than 100 cells were analyzed for each sample of γH2AX foci analysis, while more than 10,000 cells were measured for apoptosis by cytometry analysis, both are based on the methodology of the relevant published articles.
Data exclusions	No data were excluded from the analyses.
Replication	All data were obtained from at least 3 independent experiments. Data are presented as the means ± SE.
Randomization	The cultured cells were allocated into experimental groups randomly.
Blinding	The investigators were blinded for group allocation during data collection of all experiments in our study.

Reporting for specific materials, systems and methods

We require information from authors about some types of materials, experimental systems and methods used in many studies. Here, indicate whether each material, system or method listed is relevant to your study. If you are not sure if a list item applies to your research, read the appropriate section before selecting a response.

Materials & experimental systems

n/a	Involved in the study	
☐	☒	Antibodies
☐	☒	Eukaryotic cell lines
☒	☐	Palaeontology
☒	☐	Animals and other organisms
☒	☐	Human research participants
☒	☐	Clinical data

Methods

n/a	Involved in the study	
☒	☐	ChIP-seq
☐	☒	Flow cytometry
☒	☐	MRI-based neuroimaging

Antibodies

Antibodies used	Phospho-Histone H2A.X (Ser139) (20E3) Rabbit monoclonal antibody (catalog No. 9718; lot No. 13) was from Cell Signaling Technology (Beverly, MA, USA). Anti-RAC2 Goat polyclonal antibody (catalog No. ab2244; lot No. GR280949-1) was from Abcam (Cambridge, MA, USA).
Validation	Phospho-Histone H2A.X (Ser139) (20E3) Rabbit mAb (catalog No. 9718, 1:500 diluted in Antibody Dilution Buffer) was used in immunofluorescent to detect phosphorylation of H2A.X at Ser139 in 4% paraformaldehyde and iced methanol fixed human Beas-2B cells. Secondary antibody labeled with Alexa Fluor 555 was used to visualize DNA double strand breaks presented as γH2AX foci. Anti-RAC2 antibody (ab2244, 1:1000 diluted in Antibody Dilution Buffer) was used in Western blot to detect the RAC2 protein in lysates from human Beas-2B cells at 50 μg per lane. Signals were detected at the band of about 21 kDa.

Eukaryotic cell lines

Policy information about cell lines

Cell line source(s)	ATCC # CRL-9609.
Authentication	No cell line authentication was done by the authors before initiating this study.
Mycoplasma contamination	The cell line was tested negative for mycoplasma contamination by a GMyc-PCR Mycoplasma Test Kit (Yeasen, Shanghai).
Commonly misidentified lines (See ICLAC register)	None.

Flow Cytometry

Plots

Confirm that:

☒ The axis labels state the marker and fluorochrome used (e.g. CD4-FITC).

☒ The axis scales are clearly visible. Include numbers along axes only for bottom left plot of group (a 'group' is an analysis of identical markers).

☒ All plots are contour plots with outliers or pseudocolor plots.

☒ A numerical value for number of cells or percentage (with statistics) is provided.

Methodology

Sample preparation	The Beas-2B cells were collected by trypsinization, washed twice with pre-cooling PBS at 4℃, and resuspended with 250 μL binding buffer before cytometry analysis.
Instrument	BD FACSVerse.
Software	BD FACSuite.
Cell population abundance	More than 10,000 cells were analyzed for each sample.
Gating strategy	A sample without staining was used as the negative control. First, the voltage of FSC and SSC scattered light signals was adjusted to move the main cell population to the appropriate position. Then, the fluorescence value of each fluorescence channel was adjusted to a relatively low position by voltage adjustment, at this time, the voltage was used to collect samples. The samples stained with either PI or Annexin V-Alexa Fluor 647 were used to adjust fluorescence compensation between two dyes. In dual-parameter dot plot of FSC and SSC, all cells to be analyzed are gated and marked as P1, then 4 groups of PI-/Annexin V-Alexa Fluor 647-, PI+/Annexin V-Alexa Fluor 647-, PI-/Annexin V-Alexa Fluor 647+, PI+/Annexin V-Alexa Fluor 647+ in P1 were gated respectively and the ratios of these 4 groups in P1 were analyzed, among which PI-/Annexin V-Alexa Fluor 647+ group is considered as early apoptosis while PI+/Annexin V-Alexa Fluor 647+ group is considered as late apoptosis.

☒ Tick this box to confirm that a figure exemplifying the gating strategy is provided in the Supplementary Information.
